# The Surgical Stress Response and Anesthesia: A Narrative Review

**DOI:** 10.3390/jcm13103017

**Published:** 2024-05-20

**Authors:** Robert Ivascu, Ligia I. Torsin, Laura Hostiuc, Cornelia Nitipir, Dan Corneci, Madalina Dutu

**Affiliations:** 1 Department of Anesthesiology and Intensive Care, Carol Davila University of Medicine and Pharmacy, 0200021 Bucharest, Romania; iulian.ivascu@drd.umfcd.ro (R.I.); dan.corneci@umfcd.ro (D.C.); 2 Department of Anesthesiology and Intensive Care, Dr. Carol Davila Central Military Emergency University Hospital, 010242 Bucharest, Romania; ligiatorsin@gmail.com (L.I.T.); laurahostiuc1024@gmail.com (L.H.); 3 Department of Oncology, Carol Davila University of Medicine and Pharmacy, 020021 Bucharest, Romania; cornelia.nitipir@umfcd.ro; 4 Department of Oncology, Elias University Emergency Hospital, 011461 Bucharest, Romania

**Keywords:** surgical stress, neurohormonal response, immunological response, sympatho-adrenomedullary axis, hypothalamic–pituitary–adrenal axis, stress response modulation

## Abstract

The human physiological response “to stress” includes all metabolic and hormonal changes produced by a traumatic event at the micro or macro cellular levels. The main goal of the body’s first response to trauma is to keep physiological homeostasis. The perioperative non-specific adaptation response can sometimes be detrimental and can produce systemic inflammatory response syndrome (SIRS), characterized by hypermetabolism and hyper catabolism. We performed a narrative review consisting of a description of the surgical stress response’s categories of changes (neurohormonal and immunological response) followed by reviewing methods found in published studies to modulate the surgical stress response perioperatively. We described various preoperative measures cited in the literature as lowering the burden of surgical trauma. This article revises the anesthetic drugs and techniques that have an impact on the surgical stress response and proven immune-modulatory effects. We also tried to name present knowledge gaps requiring future research. Our review concludes that proper preoperative measures, adequate general anesthetics, multimodal analgesia, early postoperative mobilization, and early enteral nutrition can decrease the stress response to surgery and ease patient recovery. Anesthetics and analgesics used during the perioperative period may modulate the innate and adaptive immune system and inflammatory system, with a consecutive impact on cancer recurrence and long-term outcomes.

## 1. Introduction

The human physiological response “to stress” includes all metabolic and hormonal changes produced by a traumatic event at the micro and macro cellular levels. The stress response to tissue injury consists of the following three phases: the first “ebb phase”, a hypodynamic phase appearing the first hours after tissue trauma, is characterized by the body’s attempts to maintain homeostasis; the second “flow phase” is a hyperdynamic, hypercatabolic phase; finally, the third phase is considered a recovery phase [1]. It should be mentioned that currently, the “ebb” phase is called the early period, and the “flow” phase is called the late period [2].

The surgical stress response began to be studied in the 1920s when David Cuthbertson noticed that post-surgery, patient urine contained muscle breakdown metabolites [3]. The stress response to surgery represents a pattern of physiological and pathophysiological changes that occur in response to surgical trauma, which is influenced by the magnitude, invasiveness, and duration of surgery. The main goal of the body’s first response to trauma is to heal as fast as it can and, therefore, this response attempts to keep physiological homeostasis. The perioperative non-specific adaptation response can sometimes be detrimental and can produce systemic inflammatory response syndrome (SIRS), characterized by hypermetabolism and hyper catabolism [4]. Anesthetic drugs and techniques can attenuate and modulate this response and thus can influence the surgical outcome.

## 2. Methods: Literature Search

The goals of the present review are to present the current understanding of surgical stress response mechanisms and to discuss the results found in the literature about anesthetic drugs and techniques that modulate stress response and, implicitly, the surgical outcome. We conducted an extensive search in different databases (MEDLINE, PubMed, Scopus, Web of Science, and Embase) using the following primary search strategy: surgical stress, neurohormonal response, immunological response, perioperative stress response modulation, surgical stress and anesthesia, and anesthesia and immunomodulation and their associations. Citations up to December 2023 were included. We incorporated data from guidelines, consensus conferences, comparative studies, in vitro and in vivo studies, systematic reviews, meta-analyses, original articles, narrative review articles, and randomized controlled trials. First, we screened the abstracts, and then a full-text assessment of the articles was performed. Case reports were ruled out. We systematized results into a narrative review organized into two main parts as follows: the first explains the current knowledge on the physiology of the surgical stress response; the second is an overview of the anesthetic techniques and drugs found in the literature that have an impact on the surgical stress response and immune-modulatory effects.

## 3. Physiology of the Surgical Stress Response

**Figure 1:**
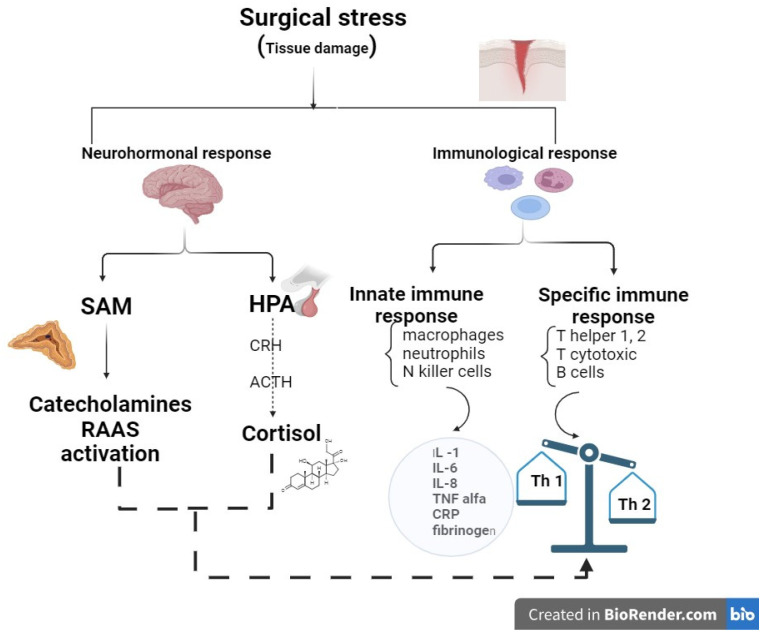
The surgical stress response components (created using BioRender.com, accessed on 4 April 2024).

The surgical stress response includes two large categories of changes as follows: a neurohormonal response and an immunological response (Figure 1).

### 3.1. The Neuro-Hormonal Response

The central nervous system receives and transmits signals to multiple organs, influencing their activity. The main pathways through which this activity is achieved were first described in 1930 by Hans Selye, namely, the sympatho-adrenomedullary axis (SAM) and the hypothalamic–pituitary–adrenal axis (HPA) [5,6,7].

### 3.2. Sympatho-Adrenomedullary Axis

Surgical trauma activates the SAM axis by generating noxious mechanical pressure stimuli that activate peripheral pain receptors, inducing nociception [8,9]. The sympathetic nervous system is activated, and information is transmitted to the cerebral cortex and thalamus through myelinated A δ fibers and unmyelinated C fibers. Nerve impulses partially reach the vasomotor area in the rostral ventrolateral medulla, activating the somato-sympathetic reflex, which has the effect of modulating vasomotor tone with an increase in peripheral resistance and heart rate [8,10]. Once the SAM axis is activated, acetylcholine neurotransmitters are released and further stimulate the adrenal gland to produce noradrenaline and adrenaline, inducing hypertension and tachycardia. Blood flow is redirected towards vital areas, decreasing kidney and gastrointestinal system supply [4]. Consequently, the renin–angiotensin–aldosterone (RAA) system is activated. Low blood flow reaching the kidney produces afferent arteriole vasoconstriction and an increase in renin secretion, which in turn modulates aldosterone secretion from the adrenal cortex. RAA overactivation induces effects on the cardiovascular, renal, and pulmonary nervous systems, with fluid retention and oliguria being commonly found in the postoperative period [4,11]. Pancreatic secretion is also changed: glucagon secretion increases, inducing glycogenolysis and hepatic gluconeogenesis, and insulin secretion decreases, leading together to a transient increase in postoperative glucose serum concentration.

### 3.3. Hypothalamic–Pituitary–Adrenal Axis

**Figure 2:**
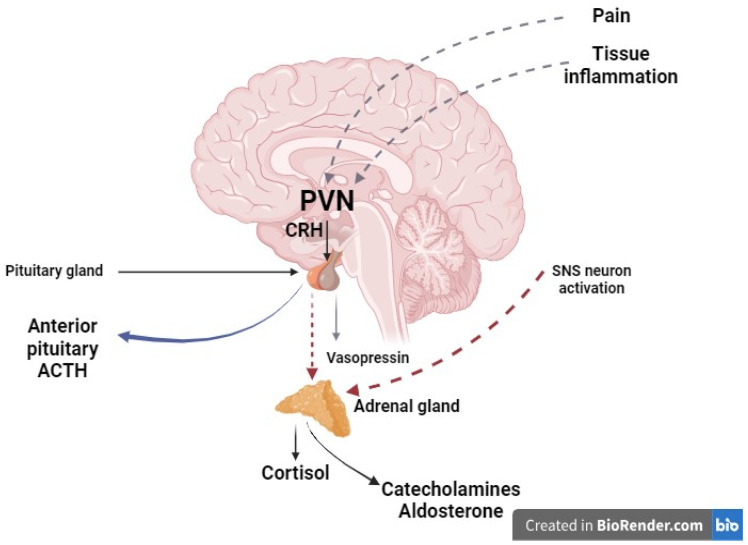
Hormonal changes in the neurohormonal surgical stress response (created using BioRender.com, accessed on 4 April 2024.

The hypothalamus projects directly into the posterior pituitary gland and controls various anterior pituitary functions. HPA axis activation originates at hypothalamus paraventricular nuclei (PVN), having a crucial role in autonomic and endocrine regulation [12,13]. Paraventricular nuclei receive and detect complex information regarding stress, emotions, and frailty through the limbic system, as well as data related to the degree of inflammation [14]. Secondary to the signal received from paraventricular nuclei, corticotropic cells from the anterior pituitary are activated and produce corticotropin-releasing hormone, which stimulates adrenocorticotropic hormone (ACTH) secretion. Through the HPA axis, ACTH promotes cortisol production from the adrenal cortex. Physiologically, ACTH and, implicitly, cortisol release, occurs based on a circadian rhythm upon which an ultradian rhythm is superimposed [6,15]. In the perioperative period, the circadian rhythm is flattened, and the ultradian rhythm increases [15]. Thus, cortisol secretion increases immediately postoperatively and reaches its maximum in 4–6 h. Although the ACTH level returns to normal 24 h following surgery, cortisol can remain at high values for up to 7 days after major surgery [16]. Cortisol effects spread overall body systems, being involved in modulation of the immunological, inflammatory, and metabolic functions (Figure 2).

### 3.4. The Immunological Response

The entire immune system developed to act against stressors, whether infectious or not, such as surgical trauma. A bimodal response is produced initially, a phase with an exaggerated inflammatory reaction—systemic inflammatory response syndrome (SIRS)—followed a few days later by compensatory anti-inflammatory response syndrome (CARS) [17,18]. In the first moment following surgical trauma, the innate immune system becomes active as the cells involved do not act against a specific antigen. The main activated cells are granulocytes (especially neutrophils and macrophages) and N killer cells. Neutrophils migrate to the injury site in a few minutes and are a marker of inflammation [19]. Likewise, macrophages immediately arrive at the trauma site, phagocytizing the destroyed tissue and releasing cytokines, which play a very important role in the inflammatory response to surgery [20].

Cytokines are a group of low-molecular-weight proteins acting on target cell receptor surfaces, modifying their protein synthesis [21]. The main proinflammatory cytokines generated are interleukin 1β (IL1β), interleukin 6 (IL6), interleukin 8 (IL8), and tumor necrosis factor-alpha (TNFα), while the main anti-inflammatory cytokines include interleukin 4 (IL4), interleukin 10 (IL10), and IL1 receptor antagonist. Secondary to the stimulating effect of cytokines (mainly IL6), important systemic changes occur, comprising the so-called acute phase response [21]. Liver proteins such as C-reactive protein (CRP), fibrinogen, and α2 macroglobulin are increased in this phase, while albumin and transferrin levels are decreased. CRP is one of the most studied inflammation mediators, which participate in dead or damaged cell clearance [22]. It increases shortly after tissue injury and has a relatively short half-life of approximately 19 h; thus, it is a good indicator of postoperative inflammation [23]. An increased value of CRP on the third to fourth post-op day is associated with postoperative complications [24].

N killer cells destroy virally infected or neoplastic cells. Surgical stress induces a decrease in cellular N killer, which is associated with the rapid growth of tumor cells and favors metastasis occurrence [25,26,27].

The specific immunological response is activated secondary to the innate response’s activation. The main cells involved are lymphocytes, which are divided into three categories as follows: T helper cells, T cytotoxic cells, and B-type cells [20]. T helper cells are divided into two categories including Th1 and Th2. Physiologically, Th1 cells destroy intracellular pathogens, while Th2 cells act against extracellular pathogens [20]. Th1 cells stimulate proinflammatory factor production, activating cellular immunity, while Th2 cells show inhibitory activity on proinflammatory factors, stimulating humoral immunity [28,29,30].

Th1 cell suppression secondary to surgical trauma occurs with a reduction in the Th1/Th2 ratio, favoring the appearance of infectious complications, such as pneumonia and wound infection [30,31,32,33]. SAM and HPA activation also produce a change in the Th1/Th2 ratio in favor of Th2 cells (Table 1).

## 4. Modulation of the Surgical Stress Response

Anesthesia enables surgery ensuring analgesia, hypnosis, and muscle relaxation as well as the perioperative body’s homeostasis [34,35]. Both anesthetic technique and anesthetics drugs used can influence the surgical stress response.

### 4.1. Preoperative Management

The surgical stress response can be diminished starting in the preoperative period. Reassurance, anxiolytics, beta-blocker use, and prolonged starvation avoidance may all lower the burden of surgical trauma.

Beta-blockers limit β-adrenergic receptor activation during surgery and therefore should theoretically lessen the amplitude of the β cell-mediated stress response and the adrenergic axis. A meta-analysis including thirty-six studies involving 319,006 patients investigated the association between administration of beta-blockers and cancer prognosis and concluded that there was no evidence to suggest an association between beta-blocker use and overall survival [36]. Despite the overall results, beta-blocker use has been associated with improved survival among patients with ovarian cancer, pancreatic cancer, and melanoma based on cancer types associated with the over proliferation of β adrenergic receptors.

α2-adrenoceptor agonists reduce central sympathetic outflow and augment endogenous opioid release in the spinal cord, influencing the descending pathways of spinal nociception. As a consequence, these effects lead to a reduction in the sympathoadrenal and cardiovascular responses to surgical stress [4]. For example, premedication with 4 mcg/kg oral dexmedetomidine has been proven to lessen the stress reaction induced by tracheal intubation, with stable hemodynamics despite a significant anesthetic-sparing effect [37]. Despite reducing preoperative anxiety and lowering cortisol levels, in patients undergoing day-case surgery [38], benzodiazepine premedication comes with an array of serious side effects, especially in the elderly, including dose-dependent sedation to respiratory depression, paradoxical reactions and antegrade amnesia, and increased pneumonia and postoperative delirium rates. European guidelines suggest avoiding benzodiazepines for premedication to prevent postoperative delirium [39].

Major surgical stress and trauma produce catabolism, and its extension is related to the magnitude of surgical stress and also the outcome. To prevent stress-related catabolism, ESPEN Guidelines on Clinical Nutrition in Surgery (2021) recommend short preoperative fasting and oral carbohydrate administration the night before and two hours before surgery as well as early postoperative enteral nutrition [40]. Moreover, in a study including elderly patients undergoing minor surgical procedures, the administration of an intraoperative low-glucose infusion attenuated fat catabolism without causing harmful hyperglycemia [41]. Immune-modulating nutrition (arginine, glutamine, omega-3 fatty acids, and nucleotides) positively modulates postsurgical immunosuppression and inflammatory responses, decreasing postoperative infectious complications and hospital length of stay [42]. ESPEN Guidelines state that there is currently no clear evidence for the sole use of these formulas enriched with immune nutrients versus standard oral nutritional supplements in the preoperative period [40].

### 4.2. Anesthetic Drugs and Techniques

Anesthetic drugs and techniques could play a key role in surgical stress response modulation. Both volatile and intravenous anesthetics alter the stress response during surgery. When facing a surgical insult, volatile anesthetic agents suppress cortisol, ACTH, growth hormone (GH), and catecholamines, to a greater extent than intravenous anesthetic agents, such as propofol combined with remifentanil [43,44]. However, propofol seems to impact the stress response mainly by inhibiting the sympathetic nervous system versus the HPA axis, as norepinephrine and glycaemia, but not ACTH and cortisol, decrease proportionally to the depth of propofol-induced anesthesia [45].

A meta-analysis of the effects of volatile agents in patients undergoing cardiac surgery showed that they could inhibit ischemic–reperfusion injury, thus exhibiting cardioprotective effects when compared with propofol TIVA (Total Intravenous Anesthesia), reducing long-term mortality [46]. However, the largest RCT among patients undergoing elective CABG including 5400 patients (randomly assigned to a volatile anesthetics group and a total intravenous anesthesia group) concluded that anesthesia using a volatile agent did not result in significantly fewer deaths in 1 year compared with total intravenous anesthesia [47].

Concerning the effects on inflammation, retrospective studies showed that volatile anesthesia could have negative effects on cancer progression [48], as it increases some tumor growth factors such as HIF1a and insulin-like growth factor. Propofol TIVA seems to decrease HIF1a and induce anti-inflammatory effects [49]. In vitro evidence confirms theory that specific anesthetic agents may impact the oncological outcome; propofol TIVA may be advantageous compared with inhalational agents, but the clinical evidence base remains relatively weak [50]. For example, halothane and isoflurane exposure increased the development of lung metastases in experimental mice with melanoma, and sevoflurane reduced NK activating receptor CD16 in primary breast cancer [51]. In contrast, there is in vitro evidence for the anti-metastatic effect of propofol TIVA; in a rat model of breast cancer metastasis, propofol TIVA, as a target-controlled infusion, inhibited the invasiveness of HeLa human cervical carcinoma cells, HT1080 human fibrosarcoma cells, HOS human osteosarcoma cells, and RPMI-7951 human melanoma cell lines [50].

Major surgery exposes the body to oxidative stress caused by reactive oxygen species generated from tissue injury and ischemia–reperfusion lesions. The degree of organ dysfunction, surgical outcome in terms of complications, and even oncological outcomes can be influenced by the equilibrium between the oxidative stress severity from surgery and the antioxidant ability of the body. From this perspective, propofol possesses anti-inflammation and anti-oxidant properties, which are organ-protective and may contribute to better postoperative analgesia compared with inhalation anesthesia [52]. Propofol has the following immune-protective effects: it is anti-inflammatory by inhibiting cyclooxygenase (COX)-2 and reducing the production of prostaglandin E2; it preserves the function of natural killer cells; it diminishes the production of cytokines; and it enhances the activation and differentiation of peripheral T-helper cells that augment cellular immunity [53,54,55]. Also, considering the impact of postoperative nausea and vomiting (PONV) on postoperative stress, TIVA with propofol has become a well-established part of multimodal strategies to reduce a patient’s risk of PONV. A meta-analysis published in 2019 showed that TIVA reduces the relative risk of PONV by 39% (95%CI 31–47%) compared with inhalational anesthesia [56].

Clinical evidence comes from retrospective studies: a retrospective analysis published in 2016 (over 7000 patients undergoing cancer surgery at a single center) comparing inhalational versus propofol TIVA anesthesia, found increased mortality at one year of almost 50% with inhalational anesthesia, increased mortality principally being seen in gastrointestinal and urological cancers [57]. The same results associated with improved survival favoring propofol TIVA use come from retrospective monocentric studies of gastric, esophageal, and breast cancer and from two meta-analyses (one included 12 studies, 10 retrospective and two prospective, with more than 21,000 patients and the other included 10 studies, nine retrospective and one prospective). Propofol TIVA seems to lead to decreased mortality and reduced postoperative pulmonary complications in patients with cancer [58,59]. A meta-analysis by Chang et al., published in 2021, included 19 retrospective observational studies of 23,489 patients undergoing surgery for various types of cancer and showed that propofol-based TIVA in cancer surgery was associated with better overall survival compared with volatile agents. This benefit was statistically significant only when TIVA was compared with desflurane but not when compared to sevoflurane or other volatile agents. The benefits were seen in patients with gastrointestinal malignancies [60]. The largest retrospective study performed by Makito et al. including 196,303 patients with gastrointestinal malignancies was published in 2020. In that retrospective study, the authors investigated the effect of TIVA and volatile agents on long-term oncological outcomes and found that overall survival (OS) and relapse-free survival (RFS) were similar between propofol-based TIVA and volatile anesthetic groups [61]. In a systematic review and meta-analysis published in 2023, comprising randomized clinical trials, the analysis between TIVA and volatile effects suggested a potential beneficial effect of propofol after the induction of anesthesia, as well as 24 h postoperatively, which, however, could not be statistically confirmed. The authors admitted that the results should be carefully evaluated since a great heterogeneity in the studies was noted [51].

There are several large prospective clinical trials on breast, colorectal, and small cell lung cancer underway, which are directly investigating the difference between propofol TIVA and sevoflurane in terms of cancer recurrence and survival in different cancer types. Whatever anesthetic technique is used; the overall goal should be to minimize the perioperative stress response.

As we emphasized in the immunological response description, NK cells target cancer cells and prevent metastasis occurrence. General anesthesia decreases NK cell cytotoxicity [62]. Ketamine and thiopental decrease NK cell activity, favoring tumor retention [43]. The combination of general and epidural anesthesia can significantly increase the NK cell count, an effect valid up to 72 h postoperatively [63].

Etomidate, an imidazole derivative, reversibly inhibits 11-β hydroxylase, thus suppressing adrenal cortisol secretion. Continuous infusion of etomidate was associated with increased mortality in critically ill patients [64], while a single induction dose decreased cortisol levels for up to 8 h without a significant effect on mortality [65]. Although it offers superior hemodynamic stability compared with other agents, etomidate use for induction in septic patients is still under debate [66].

Opioids decrease cortisol secretion, inhibiting the HPA axis at multiple levels as they reduce both CRH and ACTH secretion, but they also directly interfere with cortisol production in the adrenal gland [67]. High-dose opioid anesthesia has been used since the 1970s in cardiac surgery because of the cardiovascular stability provided. However, the use of high-dose opioids can temper metabolic and hormonal responses only at the beginning of the surgery, and patients often need mechanical ventilation in the immediate postoperative period secondary to respiratory depression [68].

Beyond their primary analgesic function, opioids exert immunosuppression effects. Opioids that can cross the blood–brain barrier and possess more immunomodulatory effects than opioids that cannot cross it; thus, morphine, fentanyl, remifentanil, methadone, and codeine have an immunomodulatory role, whereas oxycodone, tramadol, hydrocodone, and buprenorphine do not [69]. Morphine exerts dose-dependent, immunosuppressive effects, impairing monocyte and neutrophil function, NK cell-mediated cytotoxicity, and lymphocyte and macrophage proliferation [70]. Different kinds of opioids seemed to have different effects in an in vitro/in vivo model: fentanyl and sufentanyl decreased the activity of NK cells and increased the number of regulatory T cells; alfentanil decreased the activity of NK cells; and remifentanil suppressed the activity of NK cells and lymphocytic proliferation [71].

Data that emerged from the literature on the relationship between opioids and cancer recurrence indicate that opioids can modulate cancer progression by suppressing the humoral immune response and exerting pro-angiogenic effects [72]. Mu-opioid receptor, the site of mu-opioid action, is known to be overexpressed on the surface of certain cancers; therefore, opioids may influence tumor cell growth [73].

The epidemic opioid crisis is recognized as a public health emergency and the complex, and far-to-be-understood interplay between opioids and cancer has generated increasing enthusiasm for opioid-free anesthesia (OFA) techniques. Opioid-sparing strategies have emerged, embracing loco-regional techniques, nonopioid analgesics, and anti-hyperalgesics for integrated pain management. Initially, a combination of intravenous dexmedetomidine, ketamine, and lidocaine was utilized, to which newer additions like magnesium sulfate, gabapentin, dexamethasone, paracetamol, nonsteroidal anti-inflammatory drugs (NSAIDs), and cyclo-oxygenase-2 (COX-2) inhibitors were made subsequently [74]. Current knowledge regarding opioid-free or low-dose opioid anesthesia and analgesic techniques comes from its use in special populations including obesity, sleep apnea, chronic obstructive pulmonary disease, complex regional pain syndromes, acute/chronic opioid addiction, and cancer surgery [75]. The movement away from opioid usage perioperatively produced a change in thinking in analgesia management. The benefits of adapting opioid-sparing strategies in anesthesia practice comprise the following: the modulation of the neuro-inflammatory immune stress response; enhanced recovery; a reduction in overuse, prescription misuse, abuse, addiction, tolerance, and hyperalgesia as well as the prevention of sensitization; and the prevention of chronic postsurgical pain and the early return to the intended oncologic treatment and hospital discharge [74]. Three meta-analyses focusing specifically on OFA in any type of surgery, which were published up to August 2022, concluded that OFA helps prevent nausea and vomiting in a wide range of populations, decreases pain scores and opioid consumption, and improves outcomes in several surgical settings without evidence of adverse effects [76]. In a systematic review published in 2019 on opioid-free onco-anesthesia, Thota et al. concluded that the immunomodulation effects of opioids in the form of NK cell suppression found in animal studies are not demonstrable in human opioid studies, opioid effects on cancer recurrence are contradictory and not well established, and opioid effects on overall cancer survival and outcome are not causal [74]. Noteworthily, it is important to consider that it is difficult to foresee the balance between the possible immunosuppressive effect of opioids and their significant role in surgical pain control and indirect beneficial effects on immunity.

Dexmedetomidine can be a useful drug in OFA. A meta-analysis concluded that perioperative infusion of dexmedetomidine attenuates the neuroendocrine response to surgical trauma by inhibiting the release of epinephrine, norepinephrine, and cortisol. It also modulates the inflammatory and immune response by decreasing IL-6, TNF-α, and CRP and increasing IL-10, the CD4+:CD8+ ratio, and the Th1:Th2 ratio [77].

Intravenous lidocaine, as part of multimodal analgesia, produces prolonged analgesic effects; reduces opioid need; reduces inflammation, neuropathic pain, and hyperalgesia; and may positively affect wound healing. Lidocaine decreases both proinflammatory cytokine (IL-1b, IL-6 and TNF-a) and intercellular adhesion molecule (I-CAM) expression [78]. Lidocaine may also have antineoplastic effects by stimulating the NK cells of patients undergoing cancer surgery. An RCT looking at the effect of intravenous lidocaine infusion on breast cancer patients demonstrated a decrease in the postoperative expression of NETosis (which is associated with disease progression) and MMP3 [79]. A retrospective study of more than 2239 patients assessed the effect of intraoperative lidocaine (bolus injection of 1.5 mg/kg followed by continuous infusion of 2 mg/kg/h) in pancreatic surgery and suggested that intravenous lidocaine was associated with prolonged OS but not DFS [80]. It has become common to use intravenous lidocaine infusions in colorectal surgery, not to treat oncologic disease but as part of an enhanced recovery after surgery (ERAS) protocol, promoting gut motility and decreasing opioid consumption. (Table 2).

Techniques of neuraxial and regional analgesia include neuraxial (epidural/intrathecal) techniques, truncal nerve blocks, and peripheral nerve blocks. Neuraxial epidural and spinal anesthesia inhibit the transmission of afferent sensory information from surgical sites, decrease the amplitude of the HPA axis response, and decrease efferent stimulation of the liver, adrenals, and pancreas. Adrenocorticotropic hormone, cortisol, adrenaline, and GH secretions are impaired [4]. Moreover, sympathetic efferent blockade also helps in releasing fewer catecholamines. Regional anesthesia has other benefits besides contributing to analgesia and modulating surgical stress response including the following: it lowers the risk of thromboembolic events in pelvic and lower limb surgery, it improves pulmonary function and decreases pulmonary complications, it decreases cardiac complications after cardio-pulmonary bypass by interfering with the sympathetic response, and it decreases the time of paralytic ileus in abdominal surgery. When used in cardiac surgery, thoracic epidural anesthesia combined with general anesthesia can suppress the catecholamine response up to 24 h after surgery [81]. Epidural anesthesia, when combined with general anesthesia, reduces the increase in cortisol and urinary adrenaline concentrations during abdominal aortic surgery compared with general anesthesia alone [43]. Spinal anesthesia exerted serum cortisol suppression and a decrease in blood glucose concentrations compared with general anesthesia in elective abdominal, urological, and orthopedic surgery [4].

The implementation of regional anesthesia/analgesia techniques could have a positive impact on reducing cancer recurrence via several mechanisms such as the following: a reduced need for opioids or volatile agents (indirect effect); the inhibition of tumor cell growth and migration; increased activity of NK; an increased number of T-helper (Th) cells and a preserved ratio of Th1 to Th2 cells; and increased IL-4 and decreased IL-10, IL-8, and TNFalfa [71]. From this physiologic point of view, based on the powerful sympatholytic effects of regional anesthesia in addition to avoidance of the potentially detrimental immunosuppressive effects of volatile anesthetics and opioids, we would expect an improvement in cancer recurrence or outcome. However, in vitro findings are not reflected in recent in vivo studies. The results of an RCT of 400 patients that investigated the effect of combined epidural–general or general anesthesia alone in patients undergoing video-assisted thoracoscopic lung cancer resection, indicated that epidural anesthesia for major lung surgery did not improve RFS, cancer-specific survival, or OS [82]. Another large RCT including patients (*n* = 1712) undergoing major non-cardiac thoracic or abdominal surgery revealed that the effects of combined epidural–general versus general anesthesia were similar regarding mortality, cancer-specific survival, and RFS. The median follow-up time was after 5 years [83]. Two RCTs also showed a lack of benefits from paravertebral blocks in terms of cancer outcomes in patients undergoing breast cancer surgery [84].

Despite the difficulty in drawing any conclusion from the existing literature on the influence of regional anesthesia on major clinical outcomes, neuraxial and regional techniques are still important tools in the individualized anesthetic management of surgical patients. Inhibition of the stress response is greatest with central neural blockade and minimally invasive surgery.

Pain is the main part of surgical stress, leading to the activation of the hypothalamic–pituitary–adrenal axis with the next increase in the serum levels of cortisol. Untreated pain causes prolonged sympathetic stimulation which, in association with anemia and perioperative hypoxia, exposes patients with cardiac risk factors to postoperative myocardial injury/infarction (PMI) [78]. Higher levels of postoperative pain and pain distress can increase morbidity, and suboptimal postoperative analgesia is a risk factor for ongoing opioid use, opioid dependence, and persistent postsurgical pain.

Nociception monitoring might be useful not only to significantly reduce the incidence of severe postoperative pain but also to limit the stress response. The original role of the nociception monitor, which is to assess a balance between nociception caused by surgical trauma and anti-nociception due to anesthesia, may allow an assessment of surgical stress response. Currently, there are a series of commercially available devices analyzing changes in vegetative nervous system responses (heart rate and its variability, pupillary diameter, skin conductance, peripheral vasoconstriction) or the central nervous system (electroencephalographic and electromyographic patterns) and compute indexes that could guide analgesia. Although nociception by itself is difficult to measure clinically in unconscious patients, combining information from different sources with a nociception monitor was found to reflect more accurately nociception than traditionally used indirect signs [8]. Currently, no standard measurements have been defined for maximum or minimum nociception under general anesthesia, and no firm evidence has been demonstrated for any clinically relevant influence of such devices on patient outcomes [85]. Since few studies have reported associations between nociception monitor values and postoperative complications, further investigations are required to clarify associations among nociceptive monitor values, surgical stress responses, and postoperative complications to perfect multimodal general anesthesia and reduce surgical stress.

### 4.3. Surgical Techniques

Besides anesthesia techniques and drug choice, other factors influence recovery after surgery. Minimally invasive procedures, such as robotic or laparoscopic surgeries, exert less tissue damage and diminish the extent of intraoperative surgical manipulation, restraining the surgical stress response. For example, laparoscopic cystectomy versus open cystectomy resulted in lower IL-6 levels and halved the duration of postoperative SIRS [86]. In colorectal surgery, laparoscopic procedures have been associated with lower levels of IL-6, IL-1β, CRP, and TNF when compared with open surgery [87].

Furthermore, patient factors such as cancer, obesity, metabolic syndrome, diabetes, or sarcopenia contribute to the development of a hyperinflammatory state and insulin resistance. The degree of surgical insult, perioperative fasting and starvation, pain, and prolonged bed confinement further contribute to low insulin sensitivity and the surgical stress response. That is why bundle measures of prehabilitation, optimization of nutrition, standardized analgesia and anesthesia, and early mobilization were united in ERAS (early rehabilitation after surgery) protocols addressed specifically to every type of surgery [88].

### 4.4. Glucocorticoids

The anti-inflammatory benefits of perioperative glucocorticoids are still an uncertain topic. A single preoperative dose of methylprednisolone administered in elective endovascular abdominal aortic aneurysm repair reduced serum proinflammatory biomarkers (IL-6, IL-8, and CRP) and increased concentrations of the anti-inflammatory cytokine IL-10 [89]. High-dose glucocorticoid administration has been associated with a significant decrease in proinflammatory mediators (IL-6, IL-8, TNF, and CRP) and a decrease in the duration of postoperative mechanical ventilation, postoperative infection, hyperthermia, and length of hospital stay after pulmonary bypass cardiac surgery [4]. Benefits have also been shown in colorectal, hepatobiliary, and orthopedic surgeries. 

Are there any knowledge gaps in surgical stress response modulation?

A huge limitation in the current literature is represented by the impossibility of evaluating the effect of each single drug on the surgical stress response or cancer recurrence since anesthesia requires a combination of different classes of anesthetics. The difference in baseline characteristics among groups (i.e., ASA), the different concentrations of volatile anesthetics used in the clinical studies, the different duration of surgery, and the extension of surgical trauma (minimally invasive vs. open surgery) represent important confounding factors. Consequently, further studies are needed.

In vitro clinical studies showed that volatile anesthetics had substantial immunosuppressive effects. On the other hand, propofol presents anti-inflammatory and immunosuppression properties. The existing clinical evidence is mixed with some human studies, producing contrary results. More robust, prospectively collected, and controlled data are needed.

Are the findings in clinical settings that indicate the expansion of minimally invasive surgery, laparoscopic or robotic-assisted, with a secondary decrease in surgical stress amplitude consistent with propofol promoting surgical stress-induced adverse immune response better than volatile?

In the surgical setting, high-dose opioids may contribute to the inhibition of immune responses and a decreased stress response, but the clinical effects on cancer recurrence in certain types of cancer need to be better understood. We need clinical studies that evaluate the relationship between opioid-free anesthesia and the surgical stress response.

So, it is crucial for healthcare personnel to consider the possible relationships among and implications of anesthesia, perioperative stress factors, and cancer for a future better and more conscious choice of anesthetic techniques with the goal of improving cancer outcomes.

Glucocorticoid use has been associated with a reduction in serum proinflammatory biomarkers, but concerns still exist about their side effects, especially in patients with diabetes. No conclusive data exist on glucocorticoid use in elderly, frail, critically ill patients who have a reduced cortisol response influencing their outcome.

Immunomodulatory nutrition should be given to malnourished patients undergoing major cancer surgery. It is still a matter of controversy whether to use immune-modulating substrates such as arginine, omega-3 fatty acids, and nucleotides pre-, peri- or postoperatively.

There is a need for large-scale comparative studies designed specifically for laparoscopic, robotic-assisted, and open surgery to assess surgical stress response amplitude and anesthesia modulation.

## 5. Conclusions

The surgical stress response includes two large categories of changes, a neurohormonal and an immunological response, both closely interacting. The magnitude of the stress response can increase morbidity, worsen postoperative outcomes, and contribute to cancer recurrence. Anesthetics and analgesics used during the perioperative period may modulate the innate and adaptive immune system and inflammatory system, with an impact on cancer recurrence and long-term outcomes. Modulation of the amplitude of the stress response can be achieved through preoperative measures such as educational conversation and the minimization of preoperative starvation and medication. We can modulate surgical stress intraoperatively by using proper general anesthetics through intraoperative fluid management and the avoidance of intraoperative hemodynamic changes. In the postoperative period, multimodal analgesia, early postoperative mobilization, and early enteral nutrition can decrease the stress response to surgery and ease patient recovery. Despite contrasting current clinical data, it is important to increase awareness about this topic among healthcare professionals for a future conscious choice of anesthetic techniques.

## Figures and Tables

**Table 1:** Immunological changes in the surgical stress response.

Immune Response	Cell Type	Effects
Innate immune cells	Neutrophils	Act against unspecific antigen.
	Macrophages	Phagocytize destroyed tissue. Release proinflammatory cytokines (IL-6, IL-1β, TNFα, IL-8).
	N killer cells	Induce apoptosis in damaged, neoplastic, and virally infected cells.
Adaptive immune cells	T-helper 1 (Th1) lymphocytes T-helper 2 (Th2) lymphocytes	Suppression of the Th1 response. Increase in the Th2 responseTh2 > Th1 → impaired cell-mediated immunity.

**Table 2:** Overview of anesthetic drugs and the impact on the surgical stress response.

Drug	Implications
Volatile anesthetics	Suppress cortisol, ACTH, GH, and catecholamine [43,44]. Cardioprotective effects [46]. Decrease NK cell cytotoxicity [4]. Possible negative effects on cancer progression [48].
Propofol	Inhibits the sympathetic nervous system [45]. Decreases HIF1a and induces anti-inflammatory and anti-oxidant effects [49,52]. Potential beneficial effect on Th1/Th2 ratio and preserves the function of natural killer cells [51,53,54,55]. Propofol-TIVA seems to decrease mortality and cancer recurrence [57,58,59].
Etomidate	Suppresses adrenal cortisol secretion [65]. Continuous infusion associated with increased mortality in critically ill patients [64].
Ketamine	Decreases NK cell activity [43].
Opioids	Reduces CRH, ACTH, and cortisol secretion [67]. Immunosuppressive effects impairing monocyte and neutrophil function, NK cell-mediated cytotoxicity, and lymphocyte and macrophage proliferation [70,71]. Possible association with tumor recurrence after cancer surgery [72].
Dexmedetomidine	Inhibitory effects on the release of epinephrine, norepinephrine, and cortisol [77]. Decreases IL-6, TNF-α, CRP, increases IL-10, the CD4+:CD8+ ratio, and the Th1:Th2 ratio [77].
Intravenous lidocaine	Reduces inflammation and has possible antineoplastic effects [78,79].
